# Expanding the Australian Newborn Blood Spot Screening Program using genomic sequencing: do we want it and are we ready?

**DOI:** 10.1038/s41431-023-01311-1

**Published:** 2023-03-20

**Authors:** Stephanie White, Tamara Mossfield, Jane Fleming, Kristine Barlow-Stewart, Sondhya Ghedia, Rebecca Dickson, Fiona Richards, Yvonne Bombard, Veronica Wiley

**Affiliations:** 1 Faculty of Medicine and Health, Northern Clinical School, The University of Sydney, Sydney, NSW Australia; 2 Department of Clinical Genetics, Royal North Shore Hospital, Sydney, NSW Australia; 3 Genea, Sydney CBD, Sydney, NSW Australia; 4 Royal Hospital for Women, Sydney, NSW Australia; 5 Department of Clinical Genetics, Children’s Hospital, Westmead, Sydney, NSW Australia; 6 Genomics Health Services Research Program, Li Ka Shing Knowledge Institute, St. Michael’s Hospital, Unity Health Toronto, Institute of Health Policy, Management and Evaluation, University of Toronto, Toronto, Ontario Canada; 7 NSW Newborn Screening Programme, The Children’s Hospital at Westmead, Sydney, NSW Australia

**Keywords:** Health care, Population screening

## Abstract

Since the introduction of genome sequencing in medicine, the factors involved in deciding how to integrate this technology into population screening programs such as Newborn Screening (NBS) have been widely debated. In Australia, participation in NBS is not mandatory, but over 99.9% of parents elect to uptake this screening. Gauging stakeholder attitudes towards potential changes to NBS is vital in maintaining this high participation rate. The current study aimed to determine the knowledge and attitudes of Australian parents and health professionals to the incorporation of genomic sequencing into NBS programs. Participants were surveyed online in 2016 using surveys adapted from previous studies. The majority of parents (90%) self-reported some knowledge of NBS, with 77% expressing an interest in NBS using the new technology. This was significantly lower than those who would utilise NBS using current technologies (99%). Although, many health professionals (62%) felt that new technologies should currently not be used as an adjunct to NBS, 79% foresaw the use of genomic sequencing in NBS by 2026. However, for genomic sequencing to be considered, practical and technical challenges as well as parent information needs were identified including the need for accurate interpretation of data; pre-and post-test counselling; and appropriate parental consent and opt-out process. Therefore, although some support for implementing genomic sequencing into Australian NBS does exist, there is a need for further investigation into the ethical, social, legal and practical implications of introducing this new technology as a replacement to current NBS methods.

## Introduction

Newborn Blood Spot Screening (NBS) has been implemented as a public health program across the globe [1, 2]. Traditionally, the programs (tNBS) utilise biochemical and genetic technologies [3, 4] guided by the Wilson & Jungner [5] criteria for testing for treatable conditions that benefit from early intervention. In Australia, tNBS is not mandatory, but there is >99% uptake by parents, with almost 100% uniformity in the methodology and conditions tested for [6], credited with preventing serious disability and saving the lives of many newborns [6].

Debate regarding including genomic sequencing (whole genome sequencing (WGS) or whole exome sequencing (WES) analysing all variants in all genes screened- gNBS), as an addition or replacement to tNBS, centres around the potential of gNBS to identify a larger number of health conditions soon after birth, allowing earlier treatment interventions and improved pediatric outcomes [7–11]. To inform this debate, several research studies are reporting initial findings from pilot programs such as the US BabySeq project and the Australian Baby Beyond Hearing Project [12, 13]. Other studies modelling gNBS programs, such as that of Yeh et al. [14], concluded population-based gNBS for pediatric cancer predisposition syndromes is likely to reduce mortality and be cost effective [14].

However, Downie et al. [15] identified ethical, legal and social implications of gNBS: balancing the child’s and family’s best interests; the genes to be included according to their penetrance, actionability, age of onset and disease confirmation potential; the clinical validity and utility of the conditions to be included; the potential for secondary and incidental findings; and parental interest and uptake [15]. The views of stakeholders (parents, health professionals (HPs) providing NBS) are therefore important to gauge in a population where gNBS is being considered. Downie et al. [15] identified 12 studies that assessed parental or population hypothetical views on gNBS in the US, Canada and New Zealand [15] and one study which ascertained the views of HPs [16]. Although, there was a high level of interest in participation in those offered both hypothetical and actual gNBS, there were concerns about timing, parental choice regarding the scope of testing, and the impact of preferred active consent on participation rates [14].

While Australia has long been at the forefront of the implementation of tNBS, the views of stakeholders on the potential addition, or sole use, of gNBS have not been ascertained. Given this gap, an evaluation of parental, and HP, attitudes towards its use and potential implementation in Australia was undertaken to inform future debate and policy in the development of ethically and socially acceptable NBS programs: and how they might align with Wilson & Jungner criteria for tNBS [5] and modified W&J criteria [17].

## Subjects and methods

### Sample and Recruitment

Parents (>18 years) were eligible to participate if living in Australia and able to complete a survey in English. Participants were recruited through (1) posts on social media sites (e.g., Facebook pages of parents’ groups); (2) email to Genetic Alliance Support Groups; or (3) mail (contact list held by the NBS database in New South Wales) to 200 parents (children 6–12 months old). The invitation included a link to participant information and the online survey.

HPs working in tNBS and molecular genetics (clinical geneticists, genetic counsellors, pediatricians, laboratory professionals and midwives) were recruited via an emailed invitation through professional membership bodies Australian Nursing & Midwifery Federation (ANMF); NSW NBS listserv; Human Genetics Society of Australasia (HGSA) and its special interest groups. Pediatricians (selected from the NSW NBS database or working in the Sydney Children’s Hospital Network) were sent or emailed an invitation, and an advertisement was placed in the Royal Australasian College of Physicians (RACP) pediatrics online newsletter. Electronic links to the survey were also promoted by the Australian Health Practitioners Regulation Agency Facebook page and tweeted by the University of Sydney Health Sciences Twitter account. HGSA and RACP include membership from Australia and New Zealand. At the time of this study, the HGSA criteria (used to consider whether a condition should be included in tNBS) were relevant to both countries.

### Instrumentation

The online **parental survey** (Supplementary file 1) was adapted from that used for the Canadian general public in regard to gNBS (with author’s permission) [18]. In this parental survey, tNBS was described as a test for severe conditions, for which there is a good treatment option. gNBS was described as a test that additionally detects changes in a baby’s genes that might cause conditions with acknowledgement that some may not have a cure, occur in adulthood, or may be difficult to interpret. To ensure parents had the opportunity to respond based on accurate information and their own values, modifications to the parental survey (for the general public) included knowledge questions positioned before and after a brief educational element (e.g., steps of tNBS). Education and knowledge tests related to genomics, which referred to over-diagnosis, were included. Several demographic questions were added to allow for stratification of results (e.g., “Do you have a child or immediate family member with a genetic condition?”).

The online **HP survey** (Supplementary file 2) was modelled on that used to ascertain views of US HPs (with author’s permission) [16]. Two items concerning the US mandatory nature of NBS were altered to reflect the Australian context. Additional questions regarding who should disclose a particular result were changed to reflect the Australian context (e.g., general physician instead of primary care provider). These items were based on the Berg et al. [19] “binning” system with a five-point Likert scale to assess agreement/disagreement [19]. Hypothetical scenarios were used to assess the context of return of results. Opinions about changes to NBS in the setting of genomic sequencing were assessed using a five-point Likert scale (as opposed to a four-point scale in the US study) with a free text box. Multiple-choice questions regarding personal attitudes towards gNBS were also added.

In HP and parental surveys the use of whole genomic sequencing was described as the use of a whole genome rather than just testing for specific disorders.

### Data collection

Online surveys were hosted on Survey Monkey (Survey Monkey Inc, San Mateo California, USA). The parental survey was available for eight weeks (from July 2016), with two reminders sent via social media, email and letter. The HP survey was sent in a staggered fashion (May 2016 to January 2017). One reminder email was sent to genetics HPs (HGSA) and nurses and midwives (ANMF-Tasmanian branch). Participants were informed in the information sheet and survey that consent for use of their data was assumed if the survey was submitted.

### Data analysis

Survey data were analysed using Microsoft Excel and SPSSv22 (IBM Corp. Released 2013. IBM SPSS Statistics for Windows, Version 22.0. Armonk, NY: IBM Corp.). Statistical tests included paired t-test, Wilcoxon signed rank (pre-/post-survey knowledge NBS/genomic sequencing), Pearson’s chi square or Fisher exact tests and binary and multinomial logistical regression for factors associated with familiarity with NBS/genomic sequencing and attitudes to gNBS. Statistical significance was considered if *p* < 0.05.

For parental data, the sub-group that represented the greatest number of participants in each demographic variable was used as the reference group in each case. For HP data, Likert-scale responses were dichotomised into: ‘strongly agree/agree’ versus ‘unsure/disagree/strongly disagree’ and ‘of utmost importance/very important’ versus ‘moderate importance/somewhat important/not at all important’.

## Results

### Demographics

Two hundred and forty-eight Australian parents submitted a survey (85 excluded as incomplete) and 184 HP surveys were received (39 excluded due to incomplete sections). Response rate assessments were not possible given the recruitment strategies.

Overall, parents in this study had higher education levels than the general population (80% had a tertiary degree compared to 31% in the general population) with 65% earning higher than the population mean average household income (before taxes) of $113,724 (2015–2016) [20, 21]. The majority lived in NSW, had two children, and were female. Almost a third of participants reported having a relative with a genetic condition; for 51% this was their child (Table 1).

**Table 1:** Parent demographics.

Demographic variable		*N*	%
**Sex**	Male	5	3
	Female	158	97
**Age**	18–25	1	<1
	26–35	58	36
	36–45	70	43
	46–54	23	14
	55+	11	7
**Location**	ACT	12	7
	NSW	132	81
	NT	1	<1
	QLD	4	2
	SA	1	<1
	VIC	9	6
	WA	4	2
**Age of youngest child**	<1	31	19
	1–5	78	48
	6–18	26	16
	19–30	17	10
	30+	3	2
**Number of children**	1	62	38
	2	68	42
	3+	33	20
**Planning to have more children**	Yes	43	26
	No	149	91
	Unsure	27	17

Most HPs (119/145 (82%)) were female, age range 18 to > 60 years, and genetics HPs (87/145 (60%)). Over half of HPs had >10 years of experience in their field; the majority worked in the public health system (106/145 (73%)), in a metropolitan setting (103/145 (71%)) (Table 2).

**Table 2:** Health professional demographics.

	Demographic characteristic	Number of respondents *n* (%)
**Gender**	Male	26 (17.9)
	Female	119 (82.1%)
**Age**	18–30	41 (28.3)
	31–45	48 (33.1)
	46–60	42 (29.0)
	>60	14 (9.7)
**Role**	Clinical	120 (74.1)
	Research	15 (9.3)
	Teaching	5 (3.1)
	Administration	5 (3.1)
	Other	17 (10.5)
**Years working**	Less than 5 years	38 (26.2)
	5–10 years	27 (18.6)
	11–20 years	37 (25.5)
	More than 20 years	43 (29.7)
**Occupation**	Clinical Geneticist	20 (13.8)
	Genetic Counsellor	67 (46.2)
	Paediatrician	10 (6.9)
	Newborn Screening	8 (5.5)
	Midwife	18 (12.4)
	Molecular Genetics	10 (6.9)
	Other (laboratory worker)	12 (8.3)
**Sector**	Public	106 (73.1)
	Private	16 (11.0)
	Evenly in both	13 (9.0)
	Not applicable	10 (6.9)
**Geography**	Metropolitan	103 (71.0)
	Regional	14 (9.7)
	Rural	4 (2.8)
	State-wide	24 (16.6)

### Knowledge of NBS and genomic sequencing

Most parents self-reported some knowledge of NBS:71% (117/163) felt they knew a little, 18% (30/163) a lot; 7% (11/163) had heard of NBS and 3% (*n* = 5) stated they had never heard of NBS prior to completing the knowledge questions. This self-reported knowledge was reflected in an average of 91% of parents (148/163) correctly answering each of the 18 NBS knowledge questions prior to information about NBS and 93% (151/163) correctly answering each question following provision of information (z = 4.096, *p* < 0.001). Similarly, an average of 91% (148/163) of parents answered the three genetics questions (regarding over diagnosis) correctly (pre-provision of information), with knowledge improving significantly to an average of 94% (153/163) post-provision of information (z = 2.169, *p* = 0.030).

Of 145 HPs, almost all (141/143 (92%)) rated their familiarity with NBS as average or above, with those working in a clinical role significantly more likely to self-rate their familiarity as above average (χ^2^ = 16.8, *p* < 0.001). Most (116/145 (80%)) rated their familiarity with genomic sequencing as average or above, with those with less than ten years work experience significantly more likely to self-rate their familiarity as above average (χ^2^ = 15.2, *p* < 0.001). However, all midwives (*n* = 18) and individuals working in regional/rural areas (*n* = 18) rated their familiarity with genomic sequencing as average or below (Fisher 40.63, df = 2, *p* < 0.001 and Fisher 10.396, df = 2, *p* = 0.003, respectively).

### Attitudes to use of genomic sequencing in NBS

Of 163 parents, almost all (162/163 (99%)) indicated that they would be willing to participate in NBS using current technologies. However, only 77% (125/163) expressed an interest in participating in an NBS program that utilised only genomic sequencing. The number willing to participate in gNBS was therefore significantly lower than those willing to participate in tNBS (χ^2^ = 39.87, df = 1, *p* < 0.001), and lower than the actual NBS participation rates in Australia [22]. Notably, there were no significant differences in hypothetical participation rates between any of the demographic groups.

Regarding genomic sequencing being used as an adjunct to NBS, while 62% (90/141) of all HPs reported not at this time i.e., 2016, 79% (114/136) agreed it should be used within the next ten years. Midwives, laboratory workers and molecular geneticists were significantly more likely to say that genomic sequencing should be used as an adjunct now (*p* < 0.001 Exp(B) = 0.168, 95% CI 0.076–0.371) than genetics professionals, paediatricians and those working in NBS. There was no consensus as to whether genomic sequencing should be incorporated into NBS as standard practice in the future: Yes = 48/134 (36%), No = 38/134 (28%), Unsure = 48/134 (36%).

### Ethical, legal and social issues

Although NBS is not mandatory, a brief consent for collection and testing of a sample is required (opt-out). Over 85% of parents and over 90% of HPs agreed that parents should be: informed about the potential results prior to implementing gNBS (parents:158/163 (97%); HPs:141/144 (98%)); required to provide consent (parents: 152/163 (93%); HPs:135/144 (94%)); could opt-out of using the technology (parents: 140/163 (86%);HPs: 133/144 (92%)); and that data should be collected to determine whether the use of genomic sequencing in NBS was beneficial to the health of infants (parents: 160/163 (98%); HPs:139/144 (97%)). HPs also agreed that laws should be in place to protect against discrimination based on genetic information in the areas of life insurance and long-term disability (139/144 (97%)). For parents, choice was considered important (147/163 (90%)): 69% (112/163) of parents disagreed gNBS should be compulsory. In contrast, only 43% (62/144) of HPs agreed that gNBS should be optional, but highly encouraged, as for tNBS (Table 3).

**Table 3:** Parent and health professional (HP) opinions regarding the use of genomic sequencing in newborn screening (gNBS).

	Parents (*n* = 163)/ (Health professionals (*n* = 144))				
	Strongly Disagree (%)	Disagree (%)	Unsure (%)	Agree (%)	Strongly Agree (%)
gNBS should be optional (HPs only)	(10.4)	(26.4)	(20.1)	(30.6)	(12.5)
gNBS should be compulsory (Parents only)	18.4	50.3	–	16.0	15.3
Parents should be informed about potential results	1.8 (0.7)	0.6 (0.7)	−(0.7)	19.0 (25.0)	77.3 (72.9)
All sequencing results should be available to parents	1.2 (5.6)	7.4 (16.7)	−(31.3)	36.2 (25.7)	55.2 (20.8)
Parents should be able to choose types of results	3.1 (2.1)	6.7 (14.6)	−(18.1)	48.5 (43.8)	41.7 (21.5)
Parents should be required to provide consent	1.2 (1.4)	5.5 (1.4)	−3.5	33.1 (25.7)	60.1 (68.1)
Parents should have the ability to opt-out of gNBS	4.3 (2.1)	9.8 (0.7)	−(4.9)	36.8 (31.9)	49.1 (60.4)
Data should be collected to determine benefit to health of infants	0 (1.4)	1.8 (0.7)	−(1.4)	25.2 (24.3)	73.0 (72.2)
Laws against discrimination	−(1.4)	−(0.0)	−(2.1)	−(16.7)	−(79.9)

Free-text responses revealed HP concerns regarding equity of access to genomic sequencing; damage to parent-child bonding in the event of a condition of later childhood onset, incomplete penetrance or adult-onset; the need for evidence-guided use of gNBS; and the newborn’s autonomy, particularly regarding disclosure of susceptibility for adult-onset conditions. Free text responses from parents highlighted the need for explicit consent, duty of care, and a desire for research into pre-counselling requirements for gNBS (Supplementary file 3).

**Fig. 1:**
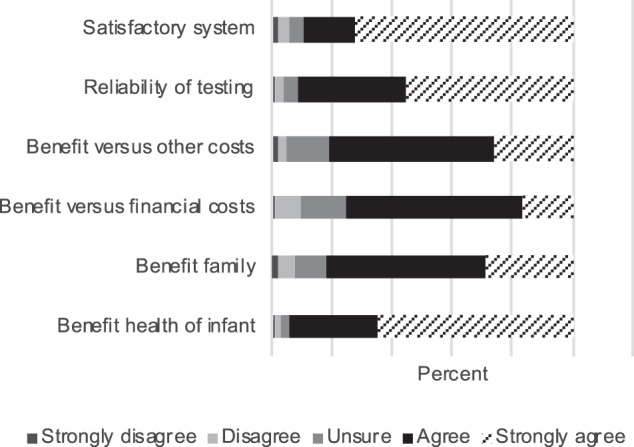
Health professionals’ opinions on criteria for inclusion of conditions in genomic newborn screening. Health professionals include clinical geneticists, genetic counsellors, pediatricians, midwives, health professionals working in molecular genetics and newborn screening.

Most HPs supported HGSA criteria [now replaced by specific jurisdiction criteria] [22] used to consider whether a condition should be included in tNBS: benefit to the baby from early diagnosis (*n* = 132/140 (94%)) and to the family (115/140 (82%)) (e.g., reproductive information); the benefit to the baby of other costs (113/140 (81%)) (e.g., psychosocial costs to the individual and family such as being “labelled”); the reliability of testing (128/140 (91%)) (e.g., there is a reasonable balance between false negative results and false positive results); and whether a satisfactory system was in operation to deal with diagnostic testing, counselling, treatment and follow-up of patients identified by the test (125/140 (89%)) (Fig. 1).

### Clinical utility and validity

Parents agreed that the doctor who orders the test should make all genomic sequencing results available to parents (149/163 (91%)) and parents should be able to choose what types of results they would like to receive (147/163 (90%)).

**Fig. 2:**
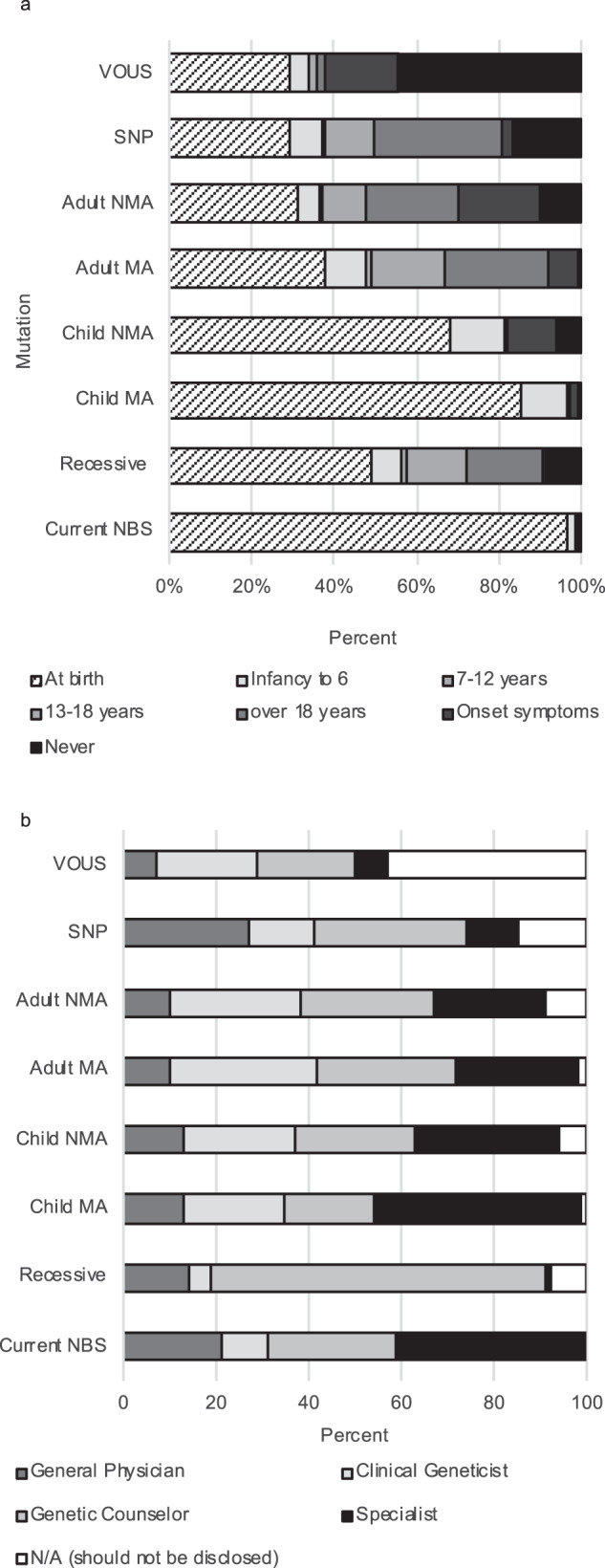
Health professionals’ opinions on timing of disclosure of genomic test results and who should disclose these results. **a** Timing of disclosure. **b** Health professional to disclose result. Health professionals include clinical geneticists, genetic counsellors, pediatricians, midwives, health professionals working in molecular genetics and newborn screening.

Of the 145 HPs, the majority felt parents should be informed at birth if their newborn carries variant/s known to cause a condition that is already tested for using tNBS methodology (125/129 (97%)); a childhood-onset disorder that is medically actionable (110/129 (85%)); or a childhood-onset disorder that is not medically actionable (88/129 (68%)). However, less than half of HPs felt parents should be informed if their newborn is a genetic carrier for a recessive condition (63/129 (49%)), an adult-onset disorder that is medically actionable (49/129 (38%)); an adult-onset disorder that is not medically actionable (40/129 (31%)); or a set of genetic markers (SNPs) known to increase their risk of an adult-onset condition (38/129 (30%)). Almost a third of HPs also indicated that finding a variant with unknown clinical implications (38/129 (30%)) or SNPs (38/129 (30%)) in a newborn should not be disclosed (Fig. 2a). Most HPs agreed specialists in the condition being disclosed should return results if a newborn has a variant known to cause a condition currently tested in tNBS (47/115 (41%)), or a childhood-onset disorder that is medically actionable (52/115 (45%)). Genetic counsellors should disclose results related to a recessive condition (83/115 (72%)) or SNP (38/115 (33%)) and genetics HPs or specialists should disclose results related to adult-onset actionable (102/115 (89%)) and non-actionable conditions (93/115 (81%)) or childhood-onset conditions (94/115 (82%)) that are not medically actionable (Fig. 2b).

**Fig. 3:**
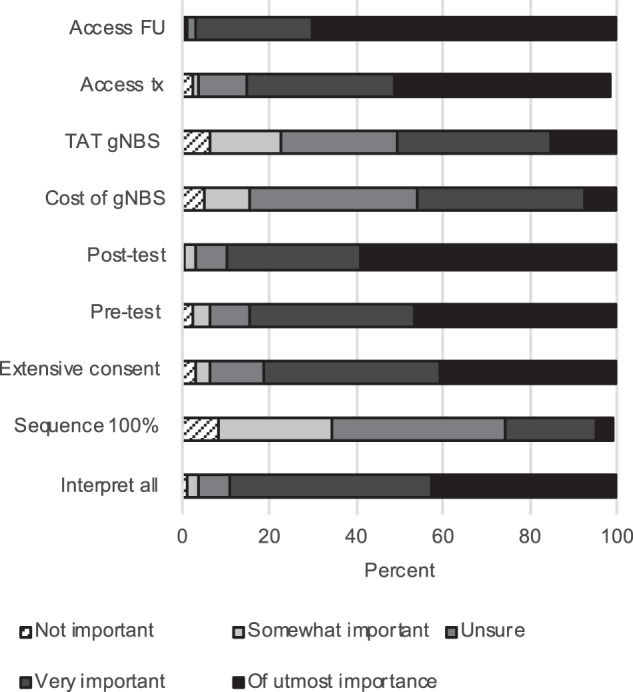
Health professionals’ opinions on potential issues of genomic newborn screening. Health professionals include clinical geneticists, genetic counsellors, pediatricians, midwives, health professionals working in molecular genetics and newborn screening.

Overall, HPs highlighted several important issues to address before considering gNBS implementation. These include the ability to accurately interpret all sequencing data (125/142 (88%)); the existence of a more extensive parental consent process (120/142 (85%)); pre- (120/142 (85%)) and post-test (127/142 (89%)) counselling for parents of infants receiving genomic sequencing; access to existing treatment for affected individuals (119/142 (84%)) and access to specialist follow-up for affected individuals (138/142 (97%)). Turn-around-time (72/142 (51%)) and cost (64/142 (46%)) were reported to be important to almost half of HPs (Fig. 3).

Additional HP concerns identified in free text responses included management of variants of uncertain significance, incidental findings, and false positives; educating HPs and the public about WGS; data privacy and storage; the inability of the current workforce to cope with population-level genomic sequencing; and parents opting-out of NBS due to the inability to predict genomic sequencing result (Illustrative quotations in Supplementary file 3).

## Discussion

This study provides the first insight into Australian parents’ and HPs’ attitudes towards gNBS. Importantly parent participants had good knowledge of the current tNBS program, which we assessed with information and quizzes. Informed public deliberation is essential for legitimate health policy, as demonstrated by Genomics England and the UK National Screening Committee [23] when they provided online information and training to 133 participants who engaged in the 2020 public dialogue deliberation regarding the implications of gNBS. Similar to the views expressed in several recent research studies, Australian parents generally supported the introduction of gNBS [15, 23–27]. Nevertheless, significantly fewer parents with good understanding were interested in participating if NBS only utilised genomics (77%), compared to current tNBS participation rates (99%), similar to a Canadian cohort [16]. This potential impact on participation rates, including reducing the number of children identified with a treatable condition and deviating from Wilson & Jungner criteria [5] that the test should be acceptable to the population (related to nature of the risk and required health education), requires consideration to ensure the continued high uptake rates of NBS. However, in the BabySeq and Baby Beyond Hearing projects - a reduction in uptake was not reported [12, 13, 15], suggesting discordance between hypothetical and real-life decisions. However, both cohorts may not be generalisable to the general population.

In comparison to parents, the majority of Australasian HPs (62%) did not believe genomic sequencing should be used in the context of NBS. However, most (79%) predicted the use of genomic sequencing as an adjunct to NBS by 2026, providing practical and technical challenges, and parent information needs, were addressed. Similarly parents and clinicians enrolled in the BabySeq study expected genomic sequencing to be more useful in 10 years [28]. Since this study was conducted, several international tNBS programs have included genomic assays for spinal muscular atrophy; a functional assay for cystic fibrosis with second-tier genomic sequencing approach; and PCR-based severe combined immuno-deficiency screening [29–31]. Screening for these conditions meets Wilson & Jungner criteria [5], testing is efficient and may be cost-effective [29, 31–33]. However, none of these approaches is the same as genomic screening of multiple genes/disorders without any previous functional assay.

Similar to other studies, the majority of Australasian HPs surveyed believe it is important to have pre- (85%) and post-test (89%) counselling (a deviation from Wilson & Jungner criteria [5]); and for affected newborns to have access to treatment (84%) and specialist follow-up (97%) [10, 16, 34] (consistent with Wilson & Jungner screening criteria [5]). Although, the majority of HPs felt it was important to be able to accurately interpret all sequencing results (88%), several acknowledged difficulties in interpretation due to limitations in current knowledge (outlined in the introduction to the HP survey). Australian parent (94%) and HP (93%) cohorts also highlighted the importance of consent, with HPs identifying the need for a more extensive consent process if gNBS programs were introduced, similar to other studies, demonstrating commitment to the ethical principle of autonomy [16, 28]. However, a more extensive consent process for genomic sequencing may reduce high participation rates if decision-making becomes too complex, as reported in a study of reasons for parental non-participation in the BabySeq project [12]. Also, the potential for gNBS to identify untreatable and/or adult-onset conditions, including cancer susceptibility syndromes and neurodegenerative conditions, may create dilemmas regarding the value of this knowledge, such as the moral authority of children, the best interests of the child/the child’s welfare, preserving the child’s autonomy to decide as an adult whether this information is wanted and maintenance of existing screening principles [28, 35, 36]. The use of a more extensive opt-in parental consent process with additional pre-test counselling would also be contrary to the current opt-out approach in Australia, Canada, and the US. Indeed, introduction of such a consent process could jeopardize the currently high community uptake, critical to support Wilson-Jungner screening criteria [5] and would introduce financial and scalability issues. It would also require an increase in trained professionals to conduct pre- and post-test counselling, which would be more time intensive than current methods [10, 24, 36–39]. Consequently, this would conflict with Wilson–Jungner criteria [5] to ensure the test is acceptable to the population and need for cost balance between screening and medical care. Consent modalities (e.g., digital approaches) that ensure engagement, equity, and simple provision of quality online information and consent may ameliorate the impact on additional workforce requirements [37, 40–42].

Whilst parents and HPs expressed cautious support and optimism for future use of gNBS, Australasian HPs identified several practical challenges. These are reflected in the literature and include adverse impacts on the relative speed and cost-effectiveness of tNBS methods; challenges with interpretation, reporting, and follow-up of genomic variants; potentially raising parental anxiety; and resource requirements, including workforce to assist parents in managing complex results [9, 10, 24, 28, 36, 39, 43–47]. Botkin and Rothwell [37] and others also recommend delaying the introduction of gNBS, highlighting unaddressed social, legal, and ethical issues [48–50]. Similarly, Ulm et al. [16] and the Australian HPs in this study felt the following issues needed to be resolved: provision and types of results to return to parents; a more extensive parental consent and appropriate opt-out process; an evaluation of the benefits to the health of infants; data management; and laws to protect individuals from genetic discrimination. These issues are not exclusive to gNBS, as they pertain to genomic sequencing in any public health program. Although Joseph et al. [51] proposed that harms could be limited and outweighed by potential benefits, they also raised concerns about maintaining control and privacy of genomic sequencing results [51]. Recent studies propose targeted newborn genomic sequencing (nGS) as an adjunct to tNBS, or as a second-tier test to identify false positive results, confirm diagnoses, and facilitate prognosis and treatment [24, 36, 39, 47].

Despite the challenges, Australian parents expressed a strong desire for all sequencing results to be available (91%) and to choose what types of results they wished to receive about their children’s health (90%). Likewise, over 90% of parents enrolled in the BabySeq study were interested in receiving results for medically actionable childhood/adulthood-onset conditions and recessive carrier status [24], similar to parents participating in the NC Nexus project [52]. However, several studies suggest parents perceive more benefits to receiving nGS results than clinicians, highlighting the need for pre-test information/counseling with discussion of risks, benefits, and utility of results [24, 25, 28].

Most Australasian HPs (97%) supported the disclosure of variants that cause conditions already tested for by tNBS at birth. The disclosure of recessive variants at birth, supported by 49% of participants, is likely to be driven by the potential for family members to benefit from this information in a reproductive context; though, the disclosure of carrier status conflicts with carrier testing policy in minors [8, 53] raising ethical and policy issues. However, the HGSA acknowledges that: carrier testing in children has not been associated with psychosocial harms; allows parents to communicate age-appropriate information to their child regarding carrier-status; whereas declining testing may cause increased parental distress, family dysfunction, uncertainty, and increased anxiety [54]. HPs preference for genetic counsellors to disclose these results is unsurprising, but again raises ethical, policy, and workforce issues regarding introducing a more extensive consent process and increasing the volume of genetic counsellor consultations [50]. Some HPs in this study also supported non-disclosure of variants of unknown significance (30%), or SNPs (30%), with three HPs commenting on potential psychosocial harm to parents of newborns. Importantly, Australasian HPs continue to support the importance of specialist care in the follow-up and treatment of newborns with a childhood-onset disorder, regardless of its actionability. These findings align with those of Ulm et al. [16] and recommendations by Berg et al. [19] that clinically actionable findings be returned, with known/presumed deleterious variants to be returned based on patient choice [16, 19]. The lack of consensus on the disclosure of adult-onset conditions suggests that Australasian HPs support the current guidelines for NBS in Australia [22], which are informed by the public health screening recommendations of Wilson and Jungner [5].

Given the availability of new technology and some discordance with established screening criteria; new or previously proposed modified framework, criteria, and principles may inform future screening paradigms [17, 55, 56]. Based on our findings, and similar to previously reported Wilson and Jungner and modified criteria, we propose several recommendations (Table 4).

**Table 4:** Recommendations for introduction of gNBS.

	Recommendations
1.	gNBS to be used as an adjunct to tNBS in the first instance.
2.	Develop consensus on results to be returned – especially adult-onset disease and SNP/VUS. Require demonstrated accurate interpretation and beneficial to health of baby.
3.	Return of results by specialists or genetics health professionals.
4.	Well-defined pre-test information and consent process (e.g., face-to-face/digital), with opt-out option.
5.	Access to specialist follow-up and treatment if required.
6.	gNBS must be cost-effective.
7.	Infrastructure/workforce in place to support change to accommodate use of gNBS.

Limitations to this study include: lack of ability to calculate a response rate; limited diversity in the parent population (higher levels of education and income than the general Australian population and almost all female); selection bias given recruitment documents were only available in English, recruitment through mothers’ groups on social media; and repetitive design of the survey to determine knowledge (which some felt was condescending). However, ensuring respondents understood the basic concepts was considered essential to validity of the data and to ensure parental views were informed. Further, as survey links were posted on Facebook and Twitter, there is the potential that responses were corrupted by bots and no process was in place to prevent this. For HPs, online recruitment of pediatricians, midwives and scientists was low. Again, no system was in place to check that it was actually HPs completing the surveys; but the risk was minimised by distributing the invitation to members through professional societies. For both groups, clarification may also have been needed regarding whether genomic sequencing would be replacing tNBS technologies or used as an adjunct. In addition, neither survey was piloted by an Australian cohort; although, both were reviewed by experts in the author list*.* Lastly, given it is six years since the surveys were conducted, it is possible that parent and HP attitudes have changed.

Future research on HP’s attitudes could include recruitment of larger numbers and a wider array of HPs to generate more generalisable results, given the critical role they will play in any gNBS implementation. In addition, a well-planned pilot research study of gNBS (in line with a 2021 UK public dialogue recommendation) could be conducted with a more ethnic mix of participants including Indigenous people, to evaluate the potential benefits of gNBS, ensure a high level of uptake, and to evaluate different approaches to consent (including type and amount of information provided about gNBS). Further, as NBS has changed significantly in the last 20 years, an exploration of the differences between the attitudes of parents whose children are older (over 18) and those whose children have been tested more recently may inform the discussion.

This is the first study to investigate a range of Australian stakeholder views on the future implementation of gNBS. Although HPs believed that we were not ready in 2016 to implement gNBS, most thought that Australia would see gNBS integrated as an adjunct to NBS in the future. Similarly, most parents (77%) appeared to be willing to participate in an NBS program that utilised only gNBS. While it is unclear if they were aware of the reduction in sensitivity and specificity associated with the technology compared to tNBS [57] the support for such participation is significantly less compared to tNBS (99%). Consequently, we believe, the stakeholder views reported here may inform a well-structured pilot of gNBS in Australia to support the deliberations of Australian policy makers [35]. In conclusion, although some support for implementing nGS into Australian NBS does exist, there is a need for further investigation into the ethical, social, legal and practical implications of introducing this new technology as a replacement or augmentation of current NBS methods.

## Supplementary information

Supplementary Files

## Data Availability

The data is available on request.
