# Supplementary material for: Expanding the Australian Newborn Blood Spot Screening Program using genomic sequencing: do we want it and are we ready?
Source: Eur J Hum Genet. 2023 Mar 20;31(6):703–11. doi: 10.1038/s41431-023-01311-1 (PMC10250371; doi:10.1038/s41431-023-01311-1)
Supplement: Supplementary file 1 — Supplementary Files [file 41431_2023_1311_MOESM1_ESM.pdf]

Supplementary file 1:

Parent survey

This online parental survey was adapted from that used for the Canadian general public in regard to gNBS (with author's permission)[17]. Modifications included the addition of knowledge questions pre- and post- provision of relevant information. Several demographic questions were added to allow for stratification of results.

Parental Attitudes to Using Whole Genome Sequencing in Newborn Screening Programs in Australia

Section A – Demographics

We would like to know a bit about you so we can better understand the people who participated in our study.

1. What year were you born?

2. What is your gender? *(Please check only one)*

   - ○ Female
   - ○ Male
   - ○ Other
   - ○ Prefer not to say

3. What state or territory do you live in? *(Please check only one)*

   - ☐ New South Wales
   - ☐ Victoria
   - ☐ Queensland
   - ☐ South Australia
   - ☐ Western Australia
   - ☐ Tasmania
   - ☐ Northern Territory
   - ☐ Australian Capital Territory

4. How many children do you have? *(Please fill in the blank)*

   ____

(a) How old is your youngest child? *(Please fill in the blank)*

Age in years ________________________________

5. What is your marital status? *(Please check only one)*

   - ☐ Single (never married, no live-in partner)
   - ☐ Married

- ☐ Common law/live-in partner
- ☐ Separated/Divorced
- ☐ Widowed
- ☐ Other *(please specify):* ____

6. Are you currently pregnant? *(Please check only one)*

- ☐ Yes
- ☐ No
- ☐ Prefer not to say

a. Is your spouse/partner currently pregnant? *(Please check only one)*

- ☐ Yes
- ☐ No
- ☐ Not applicable
- ☐ Prefer not to say

7. Do you plan to have children in the future? *(Please check only one)*

- ☐ Yes
- ☐ No
- ☐ Not sure
- ☐ Prefer not to say

8. What is the highest level of education that you have completed? *(Please check only one)*

- ☐ Did not complete high school
- ☐ Higher school certificate or equivalent
- ☐ TAFE Certificate
- ☐ Diploma
- ☐ Bachelor Degree
- ☐ Higher Degree

9. What is your annual household income (before taxes)? *(Please check only one)*

- ☐ Less than $20,000
- ☐ $20,000 – $39,999
- ☐ $40,000 - $59,999
- ☐ $60,000 - $79,999
- ☐ $80,000 - $99,999
- ☐ $100,000 - $149,999
- ☐ $150,000 or more
- ☐ Would prefer not to say

10. Do you have a child or immediate family member with a genetic condition?

○ Yes

○ No

If yes, go to Question 11 and 12. If no, go ahead to Section B).

11. If yes to Question 10, what condition does your family member have?

○ Phenylketonuria

○ Congenital Hypothyroidism

○ Congenital malformation

○ Fragile X syndrome

○ Down syndrome

○ Other….please specify____

12. What is your relationship to this family member?

They are my…

○ Child

○ Brother/sister

○ Parent

○ Other…please specify____

Section B: Introduction to Newborn Screening

We first want to find out how much people know about newborn screening, so we're going to start this part of the questionnaire by asking you some questions and then giving you some information. We want to make sure that the information we're giving you makes sense, so we'll ask you to answer some questions as you go.

1. **How much did you know about newborn screening before receiving the invitation to participate in this research study?** *(Please check only one)*

○ I had never heard of newborn screening (the heel prick test)
○ I had heard of newborn screening but I did not know anything about it
○ I knew a little about newborn screening
○ I knew a lot about newborn screening

What is newborn screening?

2-3 days after birth, parents of newborns in Australia are offered testing for several rare, serious childhood genetic conditions by taking a small sample of blood by pricking the baby's heel.

Think about the following statements in terms of whether you think each statement is either true or false. Please mark how true each statement is for you. ***(Please check one per line)***

	**True**	**False**
Newborn screening is only for babies who show signs of a health problem at birth.	☐	☐
Newborn screening can identify a condition, even when a baby seems healthy.	☐	☐
Almost all newborns in Australia go through the newborn screening process	☐	☐

The Facts

This testing is available to all parents of newborns in Australia, and is only performed with consent from parents.

Although most babies seem healthy when they are born, some of them will actually have a genetic condition. These conditions severely affect a child's health. Health problems associated with these genetic conditions may include one or more of the following:

- Sudden death or shortened life expectancy
- Physical complications related to growth, digestion, breathing and muscle strength
- Chronic pain and discomfort
- Intellectual disability

The goal of screening is to identify babies with a specific genetic condition. It might help to think of screening as a gateway that all babies pass through to identify those babies who need further investigation. Screening requires passing all babies through the gateway to try to make sure that all babies who have one of these specific conditions are identified.

How would you answer the questions now you have more information?

	**True**	**False**
Newborn screening is only for babies who show signs of a health problem at birth.	☐	☐
Newborn screening can identify a condition, even when a baby seems healthy.	☐	☐
Almost all newborns in Australia go through the newborn screening [illegible]	☐	☐

Let's see how you did on those questions.

1. Newborn screening is only for babies who show signs of a health problem at birth.

This statement is false.

All babies are offered newborn screening, even if they seem healthy.

2. Newborn screening can identify a health problem, even when a baby seems healthy.

This statement is true.

Newborn screening can identify a health problem, in babies who seem healthy.

3. Almost all newborns in Australia go through the newborn screening process.

This statement is true.

Newborn screening is done for almost all newborns in Australia.

Steps of newborn screening

The first step (and only step for most parents and newborns) in Newborn Screening is a blood test. A few drops of blood are taken from the baby's heel and collected on a card 2-3 days after birth. This blood sample is sent to a laboratory that studies these samples for signs of a certain number of conditions.

Think about the following statements in terms of whether you think each statement is either true or false. Please mark how true each statement is for you. ***(Please check one per line)***

	**True**	**False**
Most babies get a normal result on the initial test for newborn screening.	☐	☐
If a second blood test has been requested by the lab, this means that the baby has been diagnosed with a specific condition	☐	☐

If the initial test for newborn screening comes back with an abnormal result, follow-up testing may be required to confirm whether or not the baby has the specific condition.	☐	☐

The Facts

Fortunately, most babies get a normal result on the initial test.

Sometimes, a few babies will need to have a second blood test. This is usually because the first test did not give a clear result. Most babies' second tests will give normal results and your doctor will be informed.

In a very small number of babies the blood test will be abnormal. Parents will be notified by their doctor or midwife if there is a need for further tests, and if necessary, treatment of your baby.

How would you answer the questions now you have more information?

	**True**	**False**
Most babies get a normal result on the initial test for newborn screening.	☐	☐
If a second blood test has been requested by the lab, this means that the baby has been diagnosed with a specific condition	☐	☐
If the initial test for newborn screening comes back with an abnormal result, follow-up testing may be required to confirm whether or not the baby has the specific condition.	☐	☐

Let's see how you did on those questions.

1. **Most babies get a normal result on the initial test for newborn screening.**

This statement is true.

Most babies do get a normal result on the initial newborn screening test.

2. **If a second blood test has been requested by the lab, this means that the baby has been diagnosed with a specific condition**

This statement is false.

A request for a second blood test only means that the first test did not give a clear result. Most babies' second tests will give normal results and your doctor will be informed.

3. **If the initial test for newborn screening comes back with an abnormal result, follow-up testing may be required to confirm whether or not the baby has the specific condition.**

This statement is true.

Babies who get an abnormal result on the initial test may require follow-up testing depending on the condition. For example, babies with a positive test result for Cystic Fibrosis (CF) will need a sweat test after two weeks of age to determine whether the baby has CF or is only a healthy carrier. If the baby has mistakes/faults in both copies of their CFTR (CF) gene, they might not have this testing until they are 6-12 months of age.

Section C: What happens to the babies who are confirmed to have a specific condition?

Getting a diagnosis early: How much earlier?

Think about the following statements in terms of whether you think each statement is either true or false. Please mark how true each statement is for you. ***(Please check one per line)***

	**True**	**False**
Newborn screening leads to late diagnosis of childhood conditions.	☐	☐

Newborn Screening leads to early diagnosis of the conditions screened for, which allows for early treatment and intervention.	☐	☐
Some conditions have subtle symptoms that are easy to miss if a child does not go through Newborn Screening.	☐	☐

The Facts

Newborn screening leads to diagnosis of a certain number of genetic conditions in the first weeks of a baby's life.

By finding babies with a specific condition early, there are a few things that can be done. These include:

- Getting a diagnosis early
- Starting intervention early
- Learning early about the parents' chance of having another baby with the same condition

In Australia, for the conditions included in screening, this is much earlier than would be possible without Newborn Screening. This is because the condition may not have obvious symptoms, or may not affect the baby until later in childhood (for example, milder or non-classic forms of Cystic Fibrosis may have milder symptoms and so without Newborn Screening might not be identified until later childhood, adolescence or even adulthood).

How would you answer the questions now you have more information?

	True	False
Newborn screening leads to late diagnosis of childhood conditions.	☐	☐
Newborn Screening leads to early diagnosis of the conditions screened for, which allows for early treatment and intervention.	☐	☐
Some conditions have subtle symptoms that are easy to miss if a child does not go through Newborn Screening.	☐	☐

Let's see how you did on those questions.

1. Newborn screening leads to late diagnosis of childhood conditions.

This statement is false.

Newborn screening generally allows for earlier diagnosis of a childhood condition.

2. Newborn screening leads to early diagnosis of the conditions screened for, which allows for early treatment and intervention.

This statement is true. Newborn screening does allow early diagnosis and therefore early intervention and treatment.

3. Some conditions have subtle symptoms that are easy to miss if a child does not go through Newborn Screening.

This statement is true.

Some conditions do have subtle symptoms that can be easily missed by parents and doctors if a child has not undergone Newborn Screening. Newborn screening is able to identify the screened conditions, even in the absence of any obvious symptoms.

Starting medical treatments early

Think about the following statements in terms of whether you think each statement is either true or false. Please mark how true each statement is for you. ***(Please check one per line)***

	True	False

Early diagnosis through newborn screening means that medical treatments can be started right away.	☐	☐
Early medical treatment rarely improves babies’ health.	☐	☐
All of the conditions screened for by Australian Newborn Screening programs have some benefit from early treatment/intervention.	☐	☐

The Facts

When a baby is diagnosed with a condition, medical treatments can be started right away. These early medical treatments will improve the babies’ health and/or prevent serious disability or death.

Newborn Screening in Australia only screens for a set number of conditions. This is because any condition included in Newborn Screening must have some benefit to babies' health if early treatment/intervention is achieved. Therefore, Newborn Screening leads to early diagnosis, early intervention and will significantly improve the health of babies affected with a screened condition, leading to almost normal child development.

How would you answer the questions now you have more information?

	True	False
Early diagnosis through newborn screening means that medical treatments can be started right away.	☐	☐
Early medical treatment rarely improves babies’ health.	☐	☐
All of the conditions screened for by Australian Newborn Screening programs have some benefit from early treatment/intervention.	☐	☐

Let’s see how you did on the questions.

1. **Early diagnosis through newborn screening means that medical treatments can be started right away.**

This statement is true.

An early diagnosis through newborn screening does allow medical treatment to be started right away.

2. **Early medical treatment rarely improves babies’ health.**

This statement is false.

Early medical treatment for screened conditions are extremely effective in improving affected babies' health.

3. **All of the conditions screened for by Australian Newborn Screening programs have some benefit from early treatment/intervention.**

This statement is true.

Conditions are not included in Australian Newborn Screening programs unless they have established treatments/interventions that are proven to be effective.

Reproductive risk information

Think about the following statements in terms of whether you think each statement is either true or false. Please mark how true each statement is for you. ***(Please check one per line)***

	True	False
Reproductive risk information tells parents about their chance of having a baby with an [illegible]	☐	☐
Parents never get reproductive risk information through newborn screening.	☐	☐
Parents can learn about their reproductive risks through newborn screening only if their baby is affected with a screened condition	☐	☐

When newborn screening identifies a baby with an inherited condition, parents learn that they are at risk of having *another* baby with the *same* condition Newborn Screening then would give parents early reproductive risk information – that is, information about their chance of having another baby with the same condition.

How would you answer the questions now you have more information?

	True	False
Reproductive risk information tells parents about their chance of having a baby with an [illegible]	☐	☐
Parents never get reproductive risk information through newborn screening.	☐	☐
Parents can learn about their reproductive risks through newborn screening only if their baby is affected with a screened condition	☐	☐

Let's see how you did on those questions.

1. Reproductive risk information tells parents about their chance of having a baby with an inherited condition.

This statement is true.

Reproductive risk information does tell parents about their chance of having a baby with an inherited condition.

2. Parents never get reproductive risk information through newborn screening.

This statement is false.

Parents can receive reproductive risk information through Newborn Screening
if their baby is found to be affected.

3. Parents can learn about their reproductive risks through newborn screening only if their baby is affected with a screened condition.

This statement is false.

Parents of babies who are found to have a condition through screening will receive reproductive risk information. However, if a child is found to be an unaffected carrier of a condition (eg. Phenylketonuria), this will also give parents reproductive information as it means that at least one of them must also be a carrier of that condition.

<u>Section D: What happens to other babies who are identified by newborn screening?</u>

Screening tests are designed to be extra-sensitive in order to find those babies who have a condition. As a result, it is very rare for the screening tests to miss a baby with a condition.

But by screening all babies to find the few who have a condition, newborn screening also identifies some other babies. These other babies can experience a false positive result.

What are false positive results?

	True	False
A false positive result means that a baby gets a normal screening result, but is later founc [illegible]	☐	☐
A false positive result means that a baby gets an abnormal result on the initial test for newborn screening but a follow-up test shows the	☐	☐
Some of the babies who get an abnormal result on the initial test for	☐	☐

Think about the following statements in terms of whether you think each statement is either true or false. Please mark how true each statement is for you. ***(Please check one per line)***

The Facts

Most of the babies who get an abnormal result on the initial test will be confirmed to *not* have the condition when they have a follow-up test at the hospital. These are called "false positive" results.

What are false positive results?

Most of the babies who get an abnormal result on the initial test will be confirmed to *not* have a condition either through a repeated blood sample and newborn screen, or a follow-up test. These are called "false positive" results.

Things to think about:
- Parents whose babies have to go through a follow-up test will experience anxiety. This anxiety may continue for parents even after they are told their baby is actually healthy.
- Some parents may be confused by the initial abnormal result, and mistakenly believe that there is something wrong with their baby for prolonged periods of time.
- If parents misunderstand false positive results, they may view their child as vulnerable and unnecessarily limit what they can do.

How would you answer the questions now you have more information?

	True	False
A false positive result means that a baby gets a normal screening result, but is later founc [illegible]	☐	☐
A false positive result means that a baby gets an abnormal result on the initial test for newborn screening but a follow-up test shows	☐	☐
Some of the babies who get an abnormal result on the initial test for	☐	☐

Let's see how you did on those questions.

1. A false positive result means that a baby gets a normal screening result, but is later found to have a condition.

This statement is false.

A false positive result means that a baby gets an abnormal result on the first test, but a repeated blood sample and newborn screen or follow-up test shows the baby does not have a condition.

2. A false positive result means that a baby gets an abnormal result on the initial test for newborn screening but a follow-up test shows the baby does <u>not</u> have the condition.

This statement is true.

A false positive result means that a baby gets an abnormal result on the first test, but a repeated blood sample and newborn screen or follow-up test shows the baby does not have a condition.

3. Some of the babies who get an abnormal result on the initial test for newborn screening will turn out to be false positive.

This statement is true.

Some of the babies who get an abnormal result on the initial test will turn out to be false positive.

<u>Section E: How much health information do you want?</u>

Now we'd like to ask about your opinions towards health information.

Think about the following statements in terms of how you react when you are dealing with <u>health concerns</u>. Please mark how true each statement is for you. *(Please check one per line)*

	Not at all true	*A little bit*	*Somewhat true*	*Quite a bit*	*Very much true*
a. I like to gather as much information as I can before I					
b. I have difficulty making sense of information from					
c. I fear that I might find out something I don't want to					
d. I like to review information multiple times before					
e. I like to make decisions quickly.					
f. After I've made a decision, I continue to look for					
g. I think it's the doctor's job to deal with information,					
h. I feel overwhelmed by the amount of information					
i. If health information has been generated about me					
j. Just because health information about me exists					

<u>Section F: Newborn Screening with different technologies</u>

First, let's consider the technologies we use now:
Recall that newborn screening seeks to identify specific rare, serious, treatable childhood genetic conditions. Current newborn screening programs use technologies that detect specific conditions and minimize the detection of others. This is to ensure real benefits to the babies with a condition and avoid harms to other babies who are screened.

What are your views?

a. **As a parent, would you choose to participate in this type of newborn screening program?** *(Please check only one)*

Yes
No

Now let's consider the technologies we might use in the future:

With developments in technology, it is now possible to analyze a person's entire genetic code from a sample of blood. This is called **whole genome sequencing**.

Analyzing a person's genome sequence provides information about many genetic conditions at once – not only specific ones.

It is unlike other medical tests, because it generates large amounts of extra information about a person's chances of getting other genetic conditions, now or in the future.
Much of that information would be very hard for the doctors to understand, but that may change as our knowledge of genetic conditions improves.

Much of that information may also be currently hard to use to help the baby, as some of the conditions would have no treatment or cure (or may be very mild). There is often no way to know which babies will develop health problems and which will not. Because of this, all babies diagnosed with a condition will be seen by a doctor for monitoring and sometimes treatment. This is what is called over-diagnosis.

What is over-diagnosis?

Think about the following statements in terms of whether you think each statement is either true or false. Please mark how true each statement is for you. ***(Please check one per line)***

	True	False
All babies who are over-diagnosed will develop health problems.	☐	☐
Over-diagnosis can last for many years.	☐	☐
Over-diagnosis can lead to treatment of symptoms that the baby may never experience.	☐	☐

The Facts
When over-diagnosis happens, it leads to ongoing medical visits and tests to try to figure out the abnormal test results (e.g. ongoing check-ups; blood, breathing, and urine tests; x-rays). It may also lead to treatment to prevent symptoms that would never occur. Babies may be given medications that they did not need and that could be harmful.

Over-diagnosis isn't intentional or due to medical error, but it is a problem because it turns babies into patients when they may not need medical care. Doctors and parents may think the baby is sick, or be unsure whether or not the baby needs treatment. This can go on for many years.

Things to think about:
- Families may experience the anxiety and distress of believing that their child is sick.
- Children may be told they have a disability, and believe themselves to be limited by it.
- Parents may view their child as vulnerable and unnecessarily limit what they can do.

How would you answer the questions now you have more information?

	True	False

All babies who are over-diagnosed will develop health problems.	☐	☐
Over-diagnosis can last for many years.	☐	☐
Over-diagnosis can lead to treatment of symptoms that the baby may never experience.	☐	☐

Let's see how you did on those questions.

1. **All babies who are over-diagnosed will develop health problems.**

This statement is false.

Some of the babies who are over-diagnosed will not experience any health problems during their lifetime.

2. **Over-diagnosis can last for many years.**

This statement is true.

Over-diagnosis can go on for many years – or even someone's entire life.

3. **Over-diagnosis can lead to treatment of symptoms that the baby may never experience.**

This statement is true.

A baby may receive unnecessary treatment or medication throughout their lifetime due to over-diagnosis.

Newborn Screening programs in Australia do not currently sequence babies' entire genomes.

However, in the future, Newborn Screening programs could use genome sequencing instead of the tests that are currently used to screen babies.

a. **As a parent, would you choose to participate in this type of newborn screening program?** (Please check only one)

Yes
No

Please think about your reaction to the technologies discussed above. For each statement, please check the box that best describes your opinion of using this technology in newborn screening.

	Strongly disagree	*Disagree*	*Agree*	*Strongly agree*
a. Whole-genome sequencing for newborn screening should be compulsory. in the same way that newborn screening is currently.				
b. Parents should be informed prior to testing about the potential results that could come from their newborn's whole genome sequence.				
c. The doctor who orders the test should make all sequencing results available to parents.				
d. Parents should be able to choose what types of results they would like to receive from their newborn's whole genome sequence.				

e. Parents should be required to provide consent in order for their child to receive newborn screening through whole-genome sequencing.	
f. Parents should have the ability to opt out of this program in the same way that they are currently able to opt out of newborn screening.	
g. Data should be collected to determine whether this method is beneficial to the health of infants.	

We welcome your feedback and comments about this questionnaire. Please use the space below for your comments.

(You may skip this question.)

Thank you very much for completing this questionnaire!! We really appreciate your time and input!

Supplementary File 2:

Health Professional Survey

This survey was modelled on a previous questionnaire [16]. Two items concerning the US mandatory nature of NBS were altered to reflect the Australasian context. Additional questions regarding result disclosure were changed to reflect Human Genetic Society Australia's guidelines[18]. Hypothetical scenarios were used to assess context of return of results.

Information about Whole Genome Sequencing and Newborn Screening

Genetic testing methods continue to advance, and one type of testing at the forefront of this advancement is whole-genome sequencing (WGS). WGS determines a patient's genomic sequence in a single test, often for the purpose of identifying a disease-causing mutation in a patient whose clinical findings strongly suggest an underlying genetic etiology.

- Testing platforms vary, but in general WGS can identify small changes in the genome, such as single base changes and small insertions and deletions. Larger insertions, deletions, and rearrangements cannot be reliably detected.
- Most laboratories that perform WGS state that their assay will sequence over 90%, although not 100%, of the genome.
- For the purpose of identifying previously unknown sequence changes, WGS generates a greater volume of information at a lower cost than more targeted genetic testing that is currently offered.

Once sequencing is complete, the results must be compared to a human reference sequence to determine whether any differences exist. Following this, sequence changes must be interpreted using a set of laboratory-specific bioinformatics tools. Many sequence changes can be correctly classified as disease-causing or benign. Due to limitations in current knowledge of some sequence changes, many may be difficult to interpret or not able to be interpreted at all.

There has been discussion that WGS could be used as an adjunct test to current newborn screening methodology. As you may know:

- Newborn screening results are available within a baby's first week of life.
- The cost averages around AU$23.
- Newborn screening is voluntary, however almost all newborns undergo screening.

It is generally thought that if WGS were to be used for this purpose it would most likely be an adjunct test, and would not replace the current methodology being used for newborn screening. Internationally, a number of research projects are investigating multiple aspects of the use of genomic sequencing in newborns.

WGS could identify disease-causing mutations in conditions that are currently on newborn screening panels and could also identify mutations that are found in disorders not on newborn screening panels. Careful consideration would need to be given to how and if this information should be utilised. Your response to this survey will provide valuable information about professional opinion regarding this issue and could provide insight to direct further research in this area.

1. Please tick that you have read the Participant Information Statement and agree to take part in this research.

☐ Yes, I have read the Participant Information Statement and agree to take part in this research as outlined above.

2. What is your gender?
   - ☐ Male
   - ☐ Female
   - ☐ Other

3. How old are you?
   - ☐ 18 – 30
   - ☐ 31 – 45
   - ☐ 46 – 60
   - ☐ > 60 years

4. How many years have you been working in your field?
   - ☐ Less than 5 years
   - ☐ 6 – 10 years
   - ☐ 11 – 20 years
   - ☐ More than 20 years

5. In which sector do you work? (50% of your time or more)
   - ☐ Public
   - ☐ Private
   - ☐ Evenly in both
   - ☐ Not applicable

6. In what role do you spend most of your time? (50% or more)
   - ☐ Clinical
   - ☐ Research
   - ☐ Teaching
   - ☐ Administration
   - ☐ Other

7. Which of these best describes your professional role?
   - ☐ Clinical geneticist
   - ☐ Genetic counsellors
   - ☐ Paediatrician
   - ☐ Newborn Screening
   - ☐ Midwife
   - ☐ Molecular genetics
   - ☐ Other laboratory worker

8. How would you best describe your workplace geographically?
   - ☐ Metropolitan
   - ☐ Regional
   - ☐ Rural

☐ Statewide

9. How would you rate your familiarity with newborn screening and whole genome sequencing on a scale of 1 to 5 (with 1 being the least familiar and 5 being the most familiar)?

Newborn screening 1☐ 2☐ 3☐ 4☐ 5☐

Whole genome sequencing 1☐ 2☐ 3☐ 4☐ 5☐

10. How soon do you feel that this technology should be put into practice for newborn screening?

☐ Never

☐ Within the next 5 years

☐ 6 - 10 years from now

☐ 11 – 15 years from now

☐ 16 – 20 years from now

☐ More than 20 years from now

11. Do you feel that whole genome sequencing for newborn screening should be carried out now, as an adjunct to current newborn screening?

☐ Yes

☐ No

12. Please indicate your opinion regarding the importance of each of the following potential issues if whole genome sequencing were used in newborn screening

Choose from Not Important, Somewhat Important, Unsure, Very Important, Of Utmost Importance

☐ Ability to accurately interpret all sequencing results

☐ Ability to sequence closer to 100% of the genome

☐ Existence of a more extensive parental consent process

☐ Pre-test counselling for parents of infants receiving whole genome sequencing

☐ Post-test counselling for parents of infants receiving whole genome sequencing

☐ Costs of sequencing comparable to current cost of newborn screening

☐ Turn-around-time for whole genome sequencing that is comparable to turn-around-time for current newborn screening methods

☐ Access to existing treatment for affected individuals

☐ Access to specialist follow-up for affected individuals

☐ Other concerns: Please fill them in here

13. For each statement, please check the box that best corresponds with your opinion regarding using this technology as an adjunct to current newborn screening.

Choose from Strongly Disagree, Disagree, Unsure, Agree, Strongly Agree

☐ WGS should be optional but highly encouraged, in the same way that newborn screening is currently

☐ Parents should be informed prior to testing about the potential results that could come from their newborn's whole genome sequence

☐ All sequencing results should be made available to parents

☐ Parents should be able to choose what types of results they would like to receive from their newborn's whole genome sequence

☐ Parents should be required to provide consent in order for their child receive newborn screening through whole genome sequencing

☐ Parents should have the ability to opt out of this program in the same way that they are currently able to opt out of newborn screening

☐ Data should be collected to determine whether this method is beneficial to the health of infants

☐ Laws should be in place to protect against discrimination based on genetic information in the areas of life insurance and long-term disability

☐ Other concerns: Please fill them in here

14. The following statements pertain to the current Human Genetics Society of Australasia criteria that are used to assess whether a condition should be included in newborn screening. In the context of whole-genome sequencing results disclosure, to what level do you agree or disagree that each of the following criteria should be considered?

Choose from Strongly Disagree, Disagree, Unsure, Agree, Strongly Agree

☐ There is benefit to the baby from early diagnosis

☐ If there can be no benefit to the baby, there is benefit to the family (eg. reproductive information)

☐ The benefit is reasonably balanced against financial costs

☐ The benefit is reasonably balanced against other costs (eg. psychosocial costs to the individual and family such as being "labeled")

☐ The testing is reliable (eg. there is a reasonable balance between false positive results and false negative results)

☐ There is a satisfactory system in operation to deal with diagnostic testing, counselling, treatment and follow-up of patients identified by the test.

15. There are many types of results that can be found from whole genome sequencing. Sometimes these results are categorised according to their "actionability." A condition that is "medically actionable" is one for which learning about the result could influence treatment decisions. Conversely, a condition that is "not medically actionable" is one for which knowledge of the result would not change medical management.

Consider the following hypothetical results in the context of whole-genome sequencing for newborn screening. Select the responses that best correspond with your opinions regarding disclosure of results to families.

Choose from Never, At Birth, Between infancy and 6 years, Between the ages of 7 – 12, Between the ages of 13 – 18, After the age of 18, At the onset of clinical symptoms

Choose from N/A (should never be disclosed), General physician, Clinical geneticist, Genetic counsellor, Specialist in the condition being disclosed

☐ A newborn carries a mutation or set of mutations known to cause a condition that is already tested for in Australia using current newborn screening methods

☐ A newborn carries a mutation known to cause a recessive condition (the newborn is a carrier for the condition but will not develop it)

☐ A newborn carries a mutation known to cause a childhood-onset disorder that is medically actionable

☐ A newborn carries a mutation known to cause a childhood-onset disorder that is not medically actionable

☐ A newborn carries a mutation known to cause an adult-onset disorder that is medically actionable

☐ A newborn carries a mutation known to cause an adult-onset disorder that is not medically actionable

☐ A newborn carries a set of genetic markers called single-nucleotide polymorphisms (SNPs) that are known to increase his/her risk for an adult-onset condition such as diabetes or heart disease

☐ A newborn carries a genetic variant that has unknown clinical implications

16. In some states/territories, newborn screening samples can be used for medical research if the parents consent to this at the time. Please indicate the degree to which you are comfortable with the following scenarios. For the purpose of this question, you may assume the samples are de-identified.

Choose from Completely Unacceptable, Somewhat Unacceptable, Unsure, Somewhat Acceptable, Completely Acceptable

☐ No research conducted – newborn screening samples are stored after testing and not used for research

☐ Consent required – parents must consent to their newborns sample being used for research

☐ Assumed consent – consent is not sought for research, however parents can opt out of their newborn's sample being used for research if they object

☐ No consent required – research is conducted on newborn screening samples without the parents knowledge or consent

17. Do you think whole genome sequencing should be implemented as standard practice for all newborn screening in Australia in the future?

☐ Yes

☐ No

☐ Unsure

Supplementary file 3

Health Professional and Parent feedback and comments regarding NewBorn Screening programs utilising genomic sequencing (gNBS)

An online US health professional survey[16] and an online Canadian parental survey[17] (both used with the author's permission) were adapted and used to ascertain the views of Australasian parents and health professionals regarding gNBS. Multiple Choice Questions were reviewed, and selected feedback is listed below.

Theme	Example free text responses
Ethical, Legal and Social issues	
Equity of Access	"WGS in NBS should be available as an adjunct to current methods at some point. When this happens, all parents should have the option, but I don't think it should be highly encouraged in the same way NBS is now. It is a very personal choice and should be treated as such." (HP - GC)
Parent-child bonding	"I'm not sure that discovering abnormalities in the WGS in the neonatal period is the best time to look. There is a big difference between a baby with an obvious dysmorphology and a clinically normal infant who is discovered to have a genetic abnormality with possible future effects. This places enormous stress on the family. We are already seeing this in infants who have had genetic testing done antenatally or shortly after birth. A duplication or deletion may be found, causing immense stress and affecting the bond with the baby .." (HP- Pediatrician)
Autonomy of the child	"This genetic information belongs to the child not the parent. You are going to impact life insurance and health insurance with these discoveries." (HP - CG)
	"Is it really ethical to blindly screen newborns for such things? How will this be helpful to families? How will we manage the identification of adult-onset conditions and the issue of predictive testing in minors? Is it really up to the parents whether WGS should be pursued, or is this an autonomous decision that should be made by that child at a later stage in their life?" (HP - GC)
Clinical Utility, Validity and uptake	
Interpretation of results	"We need to know a lot more about the significance of variants found in the setting of population newborn screening before we should be even considering incorporating it into routine care." (HP - CG)
	"New technology with possibility of identifying many variants of uncertain significance which may cause concern or unwarranted distress. However, as more is known about these variants over

	time, I can see that WGS for NBS will become the best testing option". (HP - GC)
	"I don't see the need for compulsory WGS for every baby, BUT if it is the ONLY way to test for conditions like PKU then I would be very much in favour". (Parent)
Return of results	"There is no benefit to being diagnosed early for many conditions. We should only screen for treatable conditions where early diagnosis makes a difference". (HP - CG)
	"Concerns include the generation of results regarding adult-onset conditions and the implications this will have for newborns through their life span". (HP - GC)
Research	"The technology needs to be subject to trials/ research which is happening before being implemented". (HP - CG)
**Education and Required Infrastructure**	
Education	"Education of health professionals (both genetic and non-genetic) ordering the test." (HP - GC)
	"I think the general public needs to be able to have a conversation about the introduction of genomic technology to screening programmes. In order for this to occur, education to improve health literacy is going to be needed." (HP- GC)
	"We need this. We need to make people aware". (Parent)
Workforce	"Would be pointless doing test if there weren't enough qualified staff to discuss results." (HP - Midwife)
	"While it would be ideal for parents to choose what information they find out through WGS in newborn screening I don't believe that level of information provision and consent is feasible in the context of newborn screening. So, I think the list of conditions reported on/genes sequenced should be decided upon by an expert group and then all babies of parents who consent should be tested for the conditions on that list. Providing options is too resource intensive and informed consent is a nice ideal but difficult to achieve in reality". (HP - GC)
Informed Consent/counselling	"It is not possible to predict the possible outcomes of WGS. There would need to be a system in place to manage what information is reported, and parents should be part of this process. In order for parents to make informed decisions, access to pre-test counselling would be absolutely necessary". (HP - GC)
	"While I believe that testing for Whole Genome Sequencing would be inappropriate for most people. I now strongly believe that it should be offered as an option to parents, particularly in instances where there may be good reason for the testing to be undertaken. I would argue that strong research needs to be done as to the counselling of parents once conditions are identified so that they are informed of how genetic conditions may OR MAY NOT affect their children now and in the future. This may be a difficult task, particularly as the first point of contact for most people (GP's) are not well educated on genetic sequencing and the genetic conditions that can be identified from it. Personally, I

	believe Australia should make genome blood testing available. I have concerns". (Parent)
	"I think patients/women who have just given birth at hospitals should be treated required to provide explicit consent for things [such as] the heel prick test. The way I was approached by the nurse was as if this is the 'done thing' and there was no other option". (Parent)
Data privacy and storage	"Ethics on data privacy and storage". (HP – Molecular Genetics)
Impact on Uptake of NBS	
Uptake of gNBS	"I would be concerned that more parents would opt out of a more complicated WGS process of testing/consent etc at the expense of valuable information." (HP - GC)

HP represents Health Professional.
